# Efficient and Economic Heparin Recovery from Porcine Intestinal Mucosa Using Quaternary Ammonium-Functionalized Silica Gel

**DOI:** 10.3390/bioengineering9110606

**Published:** 2022-10-23

**Authors:** Mahmood Karimi Abdolmaleki, Anushree Das, Devang P. Khambhati, Ali Shafiee, Kayli Dimas, Carlo Alberto Velazquez, Seyed Mohammad Davachi, Sima Choubtarash Abardeh

**Affiliations:** 1 Department of Biology and Chemistry, Texas A&M International University, Laredo, TX 78041, USA or; 2 Department of Chemistry, University of Cincinnati, Cincinnati, OH 45221, USA; 3 Department of Chemistry, Cape Breton University, Sydney, NS B1P 6L2, Canada; 4 Department of Plant Protection, Ferdowsi University, Mashhad P.O. Box 9177948974, Iran

**Keywords:** heparin, amine-functionalized silica gel, cationic silica gel, quaternary ammonium-functionalized silica gel, adsorption, quaternary ammonium salt, kinetic, thermodynamic

## Abstract

Heparin, usually isolated from porcine intestinal mucosa, is an active pharmaceutical ingredient of great material value. Traditionally, diverse types of commercial resins were employed as an adsorbent for heparin retrieval from biological samples. However, more recent years have encouraged the advent of new cost-effective adsorbents to achieve enhanced heparin retrieval. Inexpensive cationic ammonium-functionalized silica gels, monodispersed with larger surface area, porosity, and higher thermal stability, were chosen to evaluate the heparin recovery yield from porcine intestinal mucosa. We demonstrated that higher positively charged and less bulky quaternary modified silica gel (e.g., QDASi) could adsorb ~28% (14.7 mg g^−1^) heparin from the real samples. In addition, we also determined suitable surface conditions for the heparin molecule adsorption by mechanistic studies and optimized different variables, such as pH, temperature, etc., to improve the heparin adsorption. This is going to be the first reported study on the usage of quaternary amine-functionalized silica gel for HEP uptake.

## 1. Introduction

**Scheme 1:**
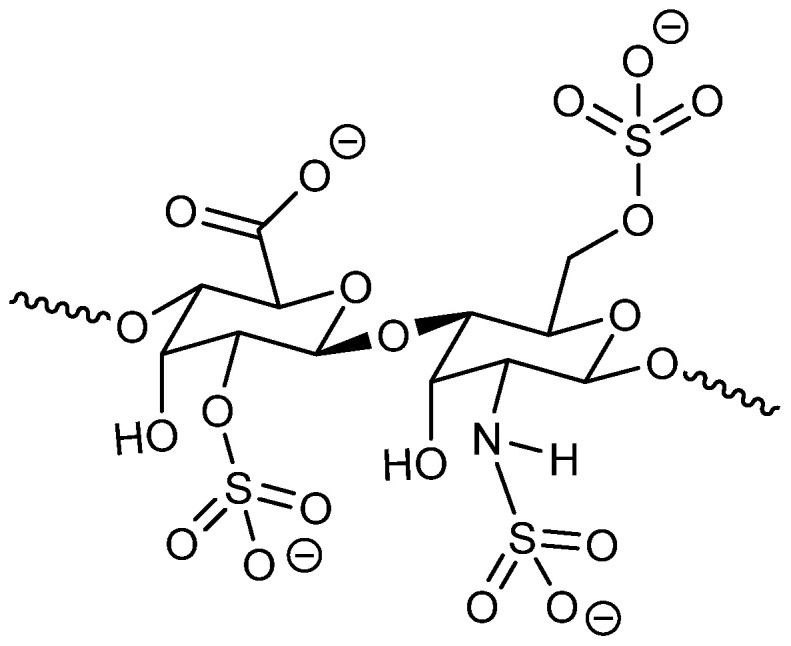
Repeating units of heparin.

Heparin (HEP) is an anticoagulant and antithrombotic agent with many pharmaceutical applications, including the treatment of acute coronary syndrome, atrial fibrillation, pulmonary embolism, and arterial thrombosis [1,2]. In addition, the antiangiogenesis, antimetastasis, and antitumor cell proliferation properties of HEP make it an extremely valuable commercial biomolecule. HEP inhibits cancer cell growth and delays the onset of Alzheimer’s disease symptoms and is applied as a soothing and anti-viral agent [3,4,5,6,7]. HEP (Scheme 1) is a polysaccharide-glycosaminoglycan that is structurally linear with varying lengths from 2000 to 40,000 kDa and is rich in sulfur [8].

HEP is typically extracted from the intestinal mucosa of pigs, even though there exist other such viable sources [9]. It is isolated through multi-step adsorption–desorption cycles after the digestion process, which depend on the adsorbent surface properties, porosity, as well as its thermodynamic and chemical stability, in order to extract the maximum possible HEP as the outcome [1,10,11,12]. However, the amount of recovery is very low and often requires multiple purifications to selectively separate HEP from other components, such as proteins, nucleic acids, etc., found in the digestion mixture, thus making the entire process laborious and unsustainable [13,14]. To date, researchers have designed and commercially employed diverse resin bead adsorbents, such as Amberlites [10], Dowex [12], Lewatit [15], and DEAE [16], to recover HEP after digestion of the animal body organs/tissues. Despite their advantages, low selectivity toward the recovery of HEP and costly manufacturing processes are the biggest challenges. Up to now, in our group, we have suggested and studied the application of several adsorbents such as ZIF-8, cross-linked spherical polycationic bead, and amine-functionalized carbon/titanium dioxide nanotube arrays on titanium foil for uptake of HEP [17,18,19]. These series of studies have demonstrated that having appropriate surface functionality is extremely helpful in increasing the adsorption efficiency of the sorbents.

Several methods for selective HEP purification through the ion-exchange technique have been reported [20]. Many of these techniques utilized primary amine or quaternary ammonium groups to make insoluble complexes selectively with HEP in the digestion mixture through electrostatic interactions between these positively charged coatings onto the surface of the adsorbent and negatively charged −COO^−^/SO_4_^2−^ groups of the HEP [2,21,22,23]. As a non-toxic and earth-abundant material, a series of amine-functionalized silica gels are commercially available at a low cost. These materials can be functionalized into cationic quaternary ammonium salts by using various simple and environment-friendly processes [24,25]. For their exhibition of adaptability in functionalization, strong chemical and thermal stability, large surface area, porosity, and monodispersity, amine-functionalized silica-gels are applied in activities such as adsorption of dyes and removal of heavy metals contaminants from the natural aquatic system [26,27,28].

**Scheme 2:**
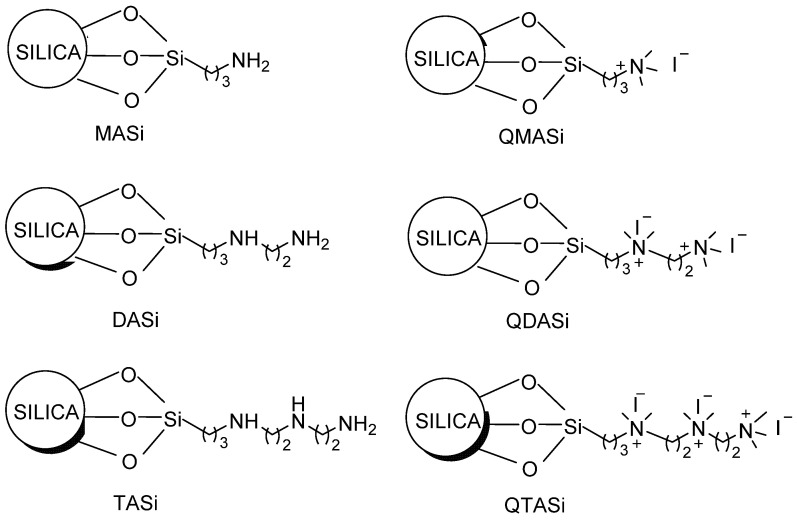
Structures of various amine-functionalized silica gel adsorbents.

In this article, we report a simple, inexpensive, and quantitative method to synthesize various cationic quaternary amine-functionalized silica gels (Scheme 2) and the retrieval of HEP from the porcine intestinal mucosa using those materials. This is going to be the first reported HEP recovery study using quaternary amine-functionalized silica gel. Results demonstrate that the higher positively charged and less bulky adsorbent surfaces (e.g., QDASi) are ideal for a higher HEP recovery. We further investigated the influence of variables such as pH, concentration of heparin, time, and adsorption process temperature to optimize HEP yield percentage. Our results show that the QDASi is reusable by simple brine wash and is stable in HEP recovery post fifth cycle. In order to compare QDASi’s anticoagulant efficacy against commercial Amberlite FPA98 Cl, we utilized measurements from sheep’s plasma. This comparison exhibits a commanding promise for quaternary ammonium-functionalized silica gel in the HEP uptake industry.

## 2. Results and Discussion

### 2.1. Synthesis and Characterization of Quaternary Ammonium-Functionalized Silica Gel

**Figure 1:**
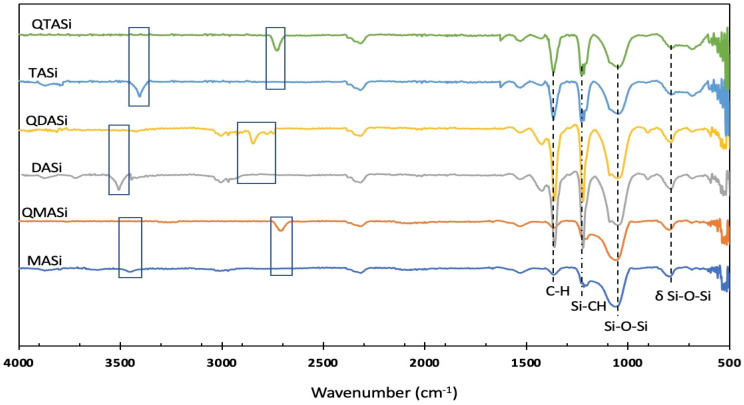
FTIR spectra of amine-functionalized silica gel and their quaternary salts (QTASi, TASi, QDASi, DASi, QMASi, and MASi).

Quaternary ammonium salts of MASi, DASi, and TASi were prepared by treating with excess methyl iodide, and the final products were characterized by IR spectra. Here, we report IR spectra of amine-functionalized silica gels and corresponding quaternary salts (Figure 1). The peaks at 787, 1043, and 1221 cm^−1^ represent δ Si-O-Si, Si-O-Si stretching, and Si-CH stretching, respectively. The peak at 1362 cm^−1^ corresponds to the bending of C-H (alkane). Quaternary salts contain additional asymmetric and symmetric C-H stretching peaks over amine-functionalized silica, which are located between 2926–2742 cm^−1^. N-H stretching peaks, for example, at 3509 cm^−1^ in DASi, are absent in QDASi due to the methyl quaternization of amine [29,30,31,32].

### 2.2. Heparin Adsorption Studies

**Figure 2:**
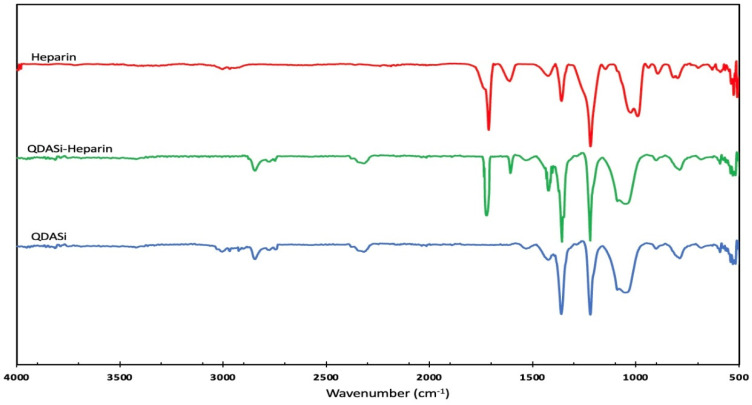
FTIR spectra of the standard heparin, QDASi, and pre-adsorbed heparin on the QDASi.

Recovering HEP from the porcine mucosa containing various other biomolecules and enzymes requires optimal parameters, including pH, contact time, dosage, and temperature to adsorb HEP on an adsorbent in harsh conditions and the desorption of the pre-adsorbed molecules to obtain pure heparin. To understand the adsorption mechanism and its commercial practicality, the adsorbent’s reusability was investigated along with kinetic and thermodynamic analysis. We performed the experiments in triplicate and evaluated the best-performing adsorbent based on the affinity toward the heparin in a pure heparin solution. For this reason, a high-concentration aqueous solution (1000 mg L^−1^) of the heparin was prepared, and 0.5 g of each quaternary ammonium-functionalized silica gel was mixed to solution, and the mixtures were stirred for 5 h at 55 °C in an incubator. The methylene blue method was utilized to calculate the adsorbed HEP [24,33]. Results (Table 1) demonstrate that the QDASi can adsorb more than three-fold of the HEP compared to QMASi in an aqueous solution. It also adsorbed 1.5-fold more HEP compared to QTASi. Although QTASi has the maximum positive charge density, its increased hydrophobicity and steric hindrance led to a lower HEP adsorption. Therefore, QDASi was chosen to investigate further HEP adsorption in the real samples obtained from the porcine intestinal mucosa. Additionally, HEP adsorption on the QDASi surface was confirmed by FTIR analysis (Figure 2). FTIR spectra of store-bought sodium heparin salt and QDASi were compared, and the C=O stretching band at 1712 cm^−1^ in QDASi-Heparin conjugate confirms the adsorption of the HEP on the QDASi surface [31,32,34].

**Figure 3:**
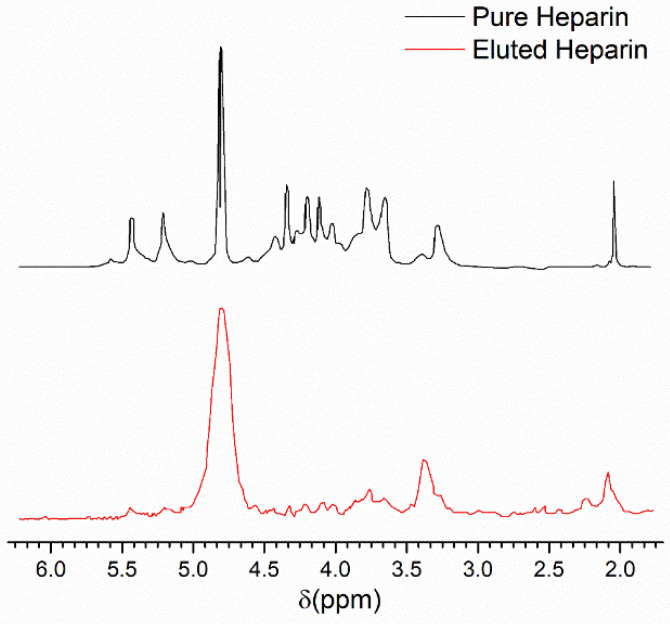
^1^H NMR spectra (400 MHz, D_2_O) of pure heparin (black) and real heparin (red), which is eluted and separated from QDASi.

NMR studies were conducted to analyze the HEP eluted and isolated from QDASi and compare it with commercial HEP. Methyl peaks in the N-acetyl glucosamine region (GlcNAc) of both isolated and commercial HEP [33] were located at 2.09 ppm and 2.08 ppm, respectively (Figure 3). This signifies effective adsorption of HEP by QDASi material. An additional peak was observed at 2.25 ppm in the HEP sample isolated from QDASi due to the presence of negatively charged darmatan sulfate [33]. Hence, crude HEP eluted from QADASi needs further purification for any pharmaceutical usage.

#### 2.2.1. Effect of pH

**Figure 4:**
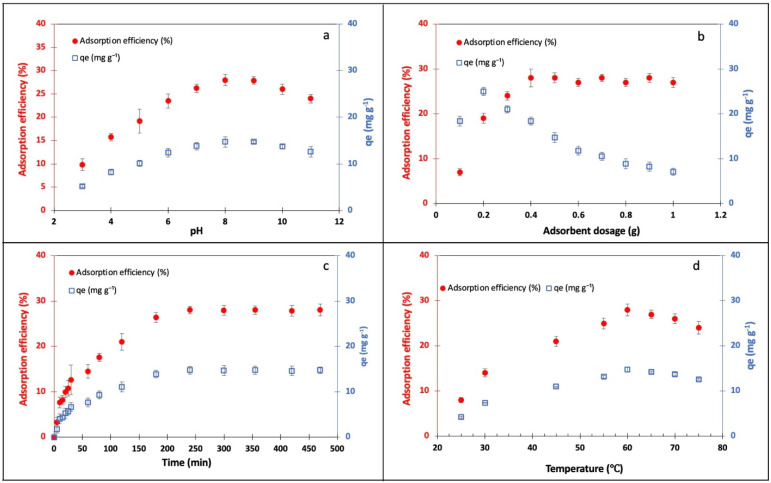
(**a**) Effect of pH (conditions: 20 mL of 1315 mg L^−1^ mucosa sample, 0.5 g of QDASi, temperature 55 °C, and 240 min of contact time); (**b**) adsorbent dosage (conditions: 25 mL of 1315 mg L^−1^ mucosa sample at pH = 8, temperature 55 °C, and 240 min of contact time); (**c**) contact time (conditions: 25 mL of 1315 mg L^−1^ real sample at pH = 8, 0.5 g of QDASi, and temperature 55 °C); and (**d**) temperature (conditions: 25 mL of 1315 mg L^−1^ real sample at pH = 8, 0.5 g of QDASi, and 300 min of contact time) of the adsorption efficiency of heparin over QDASi.

The adsorption efficiency is greatly influenced by the pH of the medium. Thus, the rate of the adsorption of HEP on QDASi was boosted through pH modulation of the real biological sample solution between 3 and 11. Results (Figure 4a) indicate that the HEP adsorption increases with an increasing range of 3 to 8 in pH. The highest adsorption with 28% efficiency and 14.7 mg g^−1^ capacity was observed at pH = 8 and then slightly decreased under more harsh alkaline conditions. Increasing the pH slightly deprotonates the HEP, which results in more negative charges and subsequently makes it more available for the active, positive sites of the QDASi. pH = 8 was considered the most optimized as QDASi neutralizes at higher pH, leading to a decrease in the absorption.

#### 2.2.2. Effect of the Adsorption Dosage

In this research, we investigated the effects of the QDASi adsorbent dosage on HEP uptake by altering the QDASi amounts from 100 to 1000 mg. Results (Figure 4b) indicate that with an increase in the QDASi dosage by up to 200 mg, adsorption capacity increased to 25 mg g^−1^. The adsorption efficiency reached a maximum of 28% at 400 mg dosage. A further increase in adsorbent dosage resulted in a capacity decrease, but the rate of adsorption remained steady. Initially, the adsorption increased due to the larger surface area with more active sites on the QDASi adsorbents. However, the adsorption capacity decreased at higher dosages. This observation occurred because of the inferring of additional components present in the mucosa sample, such as dermatan and chondroitin sulfate, which compete with the HEP and prevent it from bonding with the surface. Furthermore, a decrease in capacity was observed (25 to 7 mg g^−1^) due to the mass (m) increase in Equation (8).

#### 2.2.3. Adsorption Time and Temperature Effects

The adsorption of HEP on QDASi surfaces increases with time as the negatively charged HEP diffuses from the solution to bond on the QDASi surfaces. The results shown in Figure 4c indicate that following the increased contact time of 240 min, the adsorption efficiency (28%) as well as capacity (14.7 mg g^−1^) increased. However, the adsorption was almost stable afterward, which reveals that the QDASi is saturated under experimental conditions. Meanwhile, the solution temperature plays a vital role in the adsorption affinity. A series of experiments were performed by only varying the temperature parameter between 25 °C and 75 °C. Results (Figure 4d) demonstrate that when the temperature rises to 60 °C, the adsorption efficiency and capacity were maximized at 28% and 14.7 mg g^−1^, respectively. Solution viscosity was reduced at higher temperatures. Thus, the HEP can move faster in the solution facilitating stronger interactions with the QDASi surface. The slight reduction post 65 °C can be attributed to HEP molecule decomposition at higher temperatures.

#### 2.2.4. Kinetic and Thermodynamic Studies

**Figure 5:**
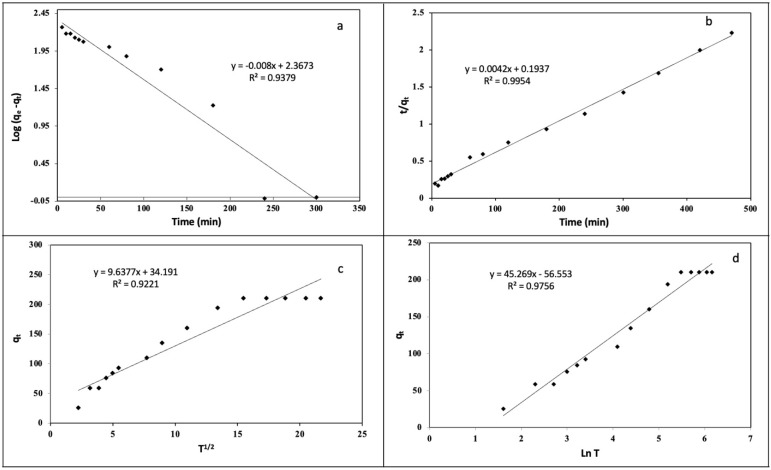
Study of the kinetics of heparin adsorption over QDASi using the pseudo-first-order (**a**); pseudo-second-order (**b**); intraparticle diffusion (**c**); and Elovich (**d**) methods.

A series of kinetic models have been used to model the adsorption phenomena of QDASi and its associated mechanisms. These include pseudo-first-order kinetic models, pseudo-second-order kinetic models, intraparticle diffusion models, and Elovich models (Figure 5 and Table 2).

Equations (1)–(4) correspond to each linearized equation of each model. Based on the assumption that the number of unoccupied sites is proportional to the number of occupied sites, the pseudo-first-order model (Equation (1)) can be constructed [35]. Using Equation (1), the slope and intercept of a linear plot of $\log\;\left(q_{e}-q_{t}\right)$ versus *t* can be derived from the value of *K*_1_ (min^−1^) and $q_{e}$, respectively, where *q_e_* (mg g^−1^) and *q_t_* (mg g^−1^) represent the adsorbed HEP at equilibrium and at any given time, respectively.

(1) $$\log\;\left(q_{e}-q_{t}\right)=\log\;q_{e}-\frac{K_{1}}{2.303}\times t$$

Using the pseudo-second-order kinetic model, it is assumed that the rate of adsorption is not determined by the adsorption of heparin but by the adsorption capacity of QDASi. Both the rate constant of the second-order equation, *K*_2_ (g mg^−1^ min^−1^), and the adsorption capacity, *q_e_* (mg g^−1^), are determined by calculating the slope and intercept of the plot *t*/*q_t_* versus *t* [36,37,38].

(2) $$\frac{t}{q_{t}}=\frac{1}{K_{2}\times q_{e}{}^{2}}+\frac{t}{q_{e}}$$

For the intraparticle diffusion model, mass transport is considered the limiting step. Using the model (Equation (3)), the rate constant of ($K_{diff},$ (mg g^−1^ min^−1^)) and the boundary thickness (*C*) are determined by plotting *q_t_* versus *t*^1/2^ [39].

(3) qt=Kdiff×t12+C

Based on Elovich’s kinetic model (Equation (4)), heterogeneous active sites are present on the sorbent, each with a varying energetic value throughout the adsorption process. With this model (Equation (4)), the Elovich constants (α and β) are determined by calculating the slope and intercept of the plot of *q_t_* versus ln(*t*) [40].

(4) $$q_{t}=\frac{1}{\beta \;\ln\;\left(\alpha \beta \right)}+\frac{1}{\beta \;\ln\;\left(t\right)}$$

Figure 5 and Table 2, which demonstrate the results of this experiment, indicate that the pseudo-second-order kinetic model best describes the adsorption of HEP onto QDASI (higher *R*^2^ and similarity of the *q_e_* theoretical and experimental). The rate-limiting step, which involves chemical adsorption, is thus the surface adsorption step. This means that HEP concentration does not affect the adsorption capacity of QDASi. The Elovich model also provides a satisfactory fit, which supports the HEP adsorption on QDASi, by assuming an exponential decrease in solute adsorption with an increase in its amount.

**Figure 6:**
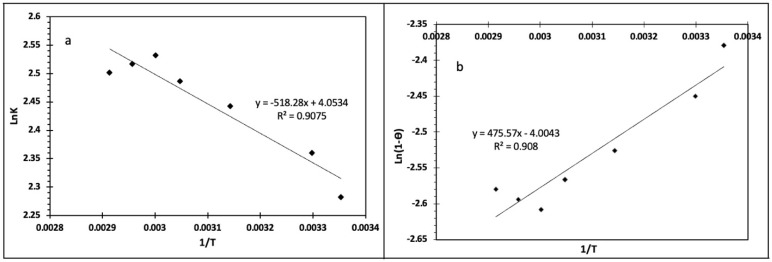
Study of the thermodynamics of heparin adsorption over QDASi using (**a**) LnK vs. 1/T, and (**b**) ln (1 − θ) versus 1/T.

A thermodynamic analysis of HEP adsorption on QDASi has been conducted using Equations (5) and (6). A plot of ln*K_c_* versus 1/*T*, and ln(1 − *θ*) versus 1/*T* (Figure 6), were used to determine the changes in entropy (Δ*S*), enthalpy (Δ*H*), Gibbs free energy (Δ*G*, calculated from Δ*G* = Δ*H* − *T*Δ*S*), and activation energy (*E_a_*) associated with the adsorption of HEP on QDASi (Table 3) [41,42,43].

*R* = 8.314 J mol^−1^ K^−1^, *T* = Temperature (*K*).

(5) $$\ln K_{c}=\frac{\Delta S}{R}-\frac{\Delta H}{RT}$$

(6) $$\ln\left(1-\theta \right)=\ln\;S^{*}+\frac{E_{a}}{RT}$$

It is evident from the positive values of Δ*H* and Δ*S* that the adsorption process is endothermic and that the HEP molecules have a high affinity for QDASi’s surface. *E_a_*’s positive value further emphatically supports the endothermic nature of the adsorption process, while Δ*G*’s negative value indicates its feasibility and spontaneity [41].

#### 2.2.5. Sorbet Reusability

**Figure 7:**
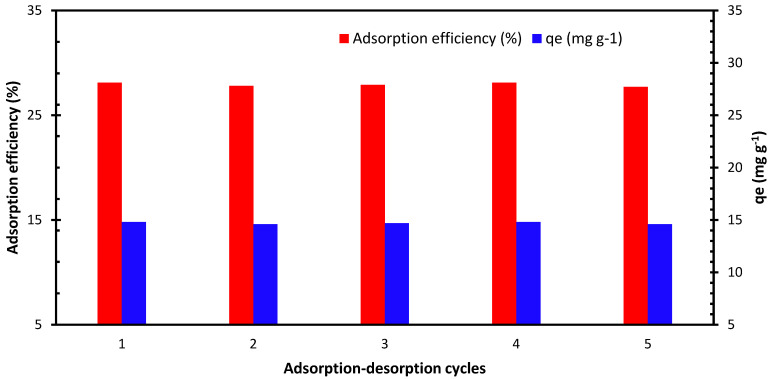
Stability test of heparin recovery over the QDASi.

QDASi has shown great performance for heparin affinity in biological samples with ~28% adsorption efficiency and 14.7 mg g^−1^ capacity under the optimized process conditions. We reused QDASi through five adsorption–desorption cycles following a harsh regeneration condition to evaluate the industrial viability of the material. QDASi regeneration involves washing it for 3 h with brine solution at 55 °C followed by Milli-Q water treatment. The regeneration process was applied for all five cycles [44]. Results (Figure 7) indicate that QDASi material is an efficient adsorbent with high stability and reusability through harsh regeneration conditions and five adsorption–desorption cycles.

#### 2.2.6. Sheep Plasma Clotting Assay

To determine the purity of HEP obtained by using QDASi, we performed a sheep plasma clotting test under optimized conditions to measure the anticoagulant potency, along with an analysis of HEP obtained using amberlite FPA98 Cl resin in accordance with the previously published method [24]. Results demonstrate that the potency of heparin eluted from QDASi (46 ± 1.8 U per g of mucosa) is very similar in performance to that eluted from commercial amberlite FPA98 Cl resin (50 ± 2.4 U per g of mucosa).

#### 2.2.7. Molecular Weight of the Extracted Heparin

The molecular weight of the extracted heparin from real samples was determined relative to the intrinsic viscosity using the Mark–Houwink–Sakurada equation (Equations (9)–(11)), where Mark-Houwink parameters K and α for heparin in water at 40 °C have been reported to be 3.16 × 10^−5^ and 0.88, respectively [45]. The weight and viscosity average molecular masses, Mw and Mv, are considered equal in dilute solutions; therefore, Mw was calculated from the same formula [46]. Heparin solutions with different concentrations from 0.05 to 0.3 g/mL in water were prepared, and the relative intrinsic viscosity, *η_rel_* was measured according to Equation (9). Using the plot of LnηrelC versus *C*, the intrinsic viscosity of polymer was 0.166 dL/g (16.587 mL/g), and the molecular weight was calculated to be 16,895 g/mol using the Mark–Houwink–Sakurada equation (Equation (11)). This value is close to the molecular weight of commercial heparin, which is around 15,500 g/mol.

## 3. Experimental

3-Aminopropyl-functionalized silica gel (MASI), 3-(Ethylenediamino)propyl-functionalized silica gel (DASI), and 3-(Diethylenetriamino)propyl-functionalized silica gel (TASI) were purchased from Sigma-Aldrich (Waltham, MA, USA). Heparin sodium salt (analytical grade), sheep plasma, hydrochloric acid (37%), calcium chloride, methanol (analytical grade), sodium hydroxide, and ethanol were purchased from VWR, US. Methyl iodide was purchased from Fisher Scientific (Waltham, MA, USA). Milli-Q water was used for sample solution preparation and the adsorption experiments. Intestinal mucosa from pork containing ∼1300 mg L^−1^ of HEP was purchased from the local market. The subtilisin enzyme was purchased from STERM company (Bellevue, WA, USA). Dow Chemical (Midland, MI, USA) sourced commercial Amberlite FPA98 Cl resin. MyBioSource (San Diego, CA, USA) supplied the Heparin ELISA kits. All chemicals were used as received.

The HEP concentration measurement was performed with SPECTROstar nano microplate reader (BMG LABTECH, Ortenberg, Germany) in both pure as well as real samples. Characterizations of the amine-functionalized silica-gels and heparin sodium salt were conducted by the Shimadzu (Kyoto, Japan) IRAffi FT-IR instrument. Functionalized silica-gels were placed in vacuum oven before and after HEP adsorption at 60 °C and 25 °C, respectively, to remove any residual solvents and moisture. The materials were then transformed into uniform powders using mortar and pestle to carry out IR experiments.

### 3.1. Synthesis (Methyl Iodide Treatment of Different Amine-Functionalized Silica)

Three methyl iodide amounts (1.0, 1.5, and 2.0 mL) were added separately to the aqueous solutions (12.5 mL) of MASi, DASi, and TASi (1 g each), respectively, at pH ~6.0 [24]. An excess methyl iodide was used for the synthesis. The reaction mixtures were filtered after a 4-h stirring at room temperature. The residues were washed with ethanol, dried under reduced pressure, and characterized by FTIR. QMASi, QDASi, QTASi (1.26, 1.43, and 1.51 g, respectively) were isolated as powders and stored in refrigerator under inert conditions and consumed during various adsorption studies.

### 3.2. Heparin Adsorption and Intrinsic Viscosity Experiments

A stock solution of HEP (1000 ppm) was prepared in Milli-Q water. The plasma of sheep and ELISA heparin kits helped evaluate the efficiency and capacity of HEP adsorption (via Equations (7) and (8)) in porcine intestinal mucosa. The details of this process are present in a prior publication [33]. The mucosa was diluted with water, and sodium chloride was added. The pH of the mixture was adjusted to 8.0 with 8 wt% sodium hydroxide solution. The mixture was then heated at 40 °C, and the protease enzyme was added while stirring the mucosa. The temperature was increased to 55 °C and held for 3 h to complete the digestion process. The enzyme was deactivated by heating the mixture at 85 °C for 30 min, and cold water (4 °C) was added. The mixture was filtered to remove any remaining large particles. QDASi (soaked in Milli-Q water) was added to this digested mucosa, and the mixture was stirred for 12 h at 55 °C. The mixture was then filtered using a Nylon cloth (60 mesh), and the recovered QDASi-Heparin complex was washed with water and sodium chloride solution (5.0 wt%) for 30 min at 50 °C to remove the unbound impurities. The complex was filtered again, and the heparin was desorbed by three steps of washing with brine at 50 °C for 3, 1, and 1 h, followed by collecting the filtrate at each washing step. The filtrates were combined, and the eluted heparin was precipitated by the addition of ethanol, followed by centrifugation. The resulting heparin was dried in a vacuum oven at 65 °C. All the measurements were completed using the same batch of mucosa sample.

(7) $$Adsorption\;efficiency\;\left(\%\right)=\frac{\left(C_{0}-C_{e}\right)}{C_{0}}\times 100$$

(8) $$Adsorption\;capacity\;\left(q_{e}\right)=\frac{\left(C_{0}-C_{e}\right)}{m}\times V$$

where *C*_0_ and *C_e_* (mg L^−1^) are the initial and equilibrium heparin concentrations (measured with the ELISA kit), *V* (L) is the volume of the mucosa solution used for heparin adsorption, and m is the mass of the adsorbent used (g).

The intrinsic viscosity of the heparin was measured in dilute DI water solution at 40 °C using a minivisc 3000 series (Spectro Scientific, Chelmsford, MA, USA) by measuring relative viscosity from Equation (9) using kinematic viscosities, implementing the Kraemer equation (Equation (10)) and plotting LnηrelC versus concentration (*C*); the intrinsic viscosity and molecular weight were calculated using Mark–Houwink–Sakurada equation (Equation (11)).

(9) ηrel=ηsampleηDI water

(10) $$\frac{{Ln\eta }_{rel}}{C}=\left[\eta \right]-K^{\prime }{\left[\eta \right]}^{2}C$$

(11) $$\left[\eta \right]=K\cdot M_{w}^{\alpha }$$

## 4. Conclusions

In this research, we investigate heparin recovery by quaternary ammonium-functionalized silica gel. To the best of our knowledge, this is the first example of heparin recovery by positively charged silica gel. We have synthesized a series of quaternary ammonium-functionalized silica gel and shown that QDASi is a better candidate for heparin recovery with up to 28% of adsorption from the real sample. High charge density, reduced hydrophobicity, and steric crowding around the active sites of QDASi can be attributed to such results. The mechanistic adsorption study demonstrates that the adsorption process obeys the pseudo-second-order kinetic model and is thermodynamically endothermic and feasible. Further investigation on optimal heparin adsorption was conducted by varying parameters such as temperature, pH, and dosage. We also measured the molecular weight of the heparin, which was close to commercial heparin.

## Figures and Tables

**Table 1:** Adsorption efficiencies and capacities of amine-functionalized silica-gel and their quaternary salts on pure HEP standard solution.

Material	Adsorption Efficiency (%)	Adsorption Capacity (mg g^−1^)
Silica-gel	<5	<3
MASi	<5	<3
DASi	<5	<3
TASi	<5	<3
QMASi	18	9.5
QDASi	59	31
QTASi	38	20

**Table 2:** Parameters calculated from different kinetic study models of heparin adsorption on QDASi.

Model	Parameter	Value
**Pseudo-first-order**	$K_{1}$	0.0186
	$q_{e\;\left(cal\right)}$	236.065
	$R^{2}$	0.9379
**Pseudo-second-order**	$K_{2}$	9.326 × E^−5^
	$q_{e\;\left(cal\right)}$	235.312
	$R^{2}$	0.9954
**Intraparticle Diffusion**	$K_{diff}$	9.637
	C	34.190
	$R^{2}$	0.9221
**Elovich**	β	0.0220
	α	1.333
	$R^{2}$	0.9756
	$q_{e\;\left(exp\right)}$	210.526

**Table 3:** Calculated thermodynamic parameters for the heparin adsorption onto QDASi.

**ΔH° (J/mol K)**	**ΔS° (KJ/mol)**	**E_a_ (KJ/mol)**	**Temperature (K)**							
			298.15	303.15	318.15	328.15	333.15	338.15	343.15	348.15
3.59	31.41	3.29	**ΔG° (KJ/mol)**							
			−9.362	−9.519	−9.990	−10.305	−10.462	−10.619	−10.776	−10.933

## Data Availability

The graphic abstract was created with BioRender.com (accessed on 22 September 2022).

## Abbreviations

HEP	Heparin
MASi	3-Aminopropyl-functionalized silica gel
DASi	3-(Ethylenediamino)propyl-functionalized silica gel
TASi	3-(Diethylenetriamino)propyl-functionalized silica gel
QMASi	Quaternarized MASi
QDASi	Quaternarized DASi
QTASi	Quaternarized TASi
